# Observations on Remittent Fever as It Occurs in the Southern Part of Alabama

**Published:** 1846-09

**Authors:** 


Art. VI.—*Observations on Remittent Fever as it occurs in the Southern part of Alabama.* By William M. Boling, M.D., of Montgomery, Alabama. From the American Journal of Medical Sciences for April, 1846. 8vo. pp. 56.

Dr. Boling is known to our readers as an industrious and judicious writer, zealously bent upon the advancement of medical science, and ready and able to communicate to his professional brethren the results of his professional experience. His various papers published in this Journal and elsewhere show him to be a man of close and patient observation, of sound judgement, excellent powers of discrimination, and independence of thought and opinion. This tract appeared a few months since in one of our contemporaries, and copies of it have been done up in pamphlet form for gratuitous distribution among the author's friends. It has already attracted an unusual share of attention, having been referred to frequently by Dr. Clymer in his late treatise on fever, and noticed in complimentary terms by many of the medical journals of the United States. Among other forms of fever not so commonly observed, Dr. Boling describes the *comatose* remittent, of which he says—

"Generally after a slight chill, high febrile excitement comes on, with flush of the face and general redness, with fulness and plumpness of the surface of the body; full and firm pulse; throbbing of the carotids; and deep and complete stupor. The respiration is slow, deep, and sometimes stertorous. Pupils frequently dilated, rarely contracted. The patient remains in this condition for a longer or shorter period, according to the type of the fever, tertain or quotidian. Gradually the stupor wears off, and for several hours of a remission scarcely any appearance of danger may be present. Again, at the regular period, but frequently anticipating by several hours, the same train of symptoms set in with greater severity, to be followed by a less perfect remission, and so with each paroxysm, till a fatal termination takes place, or a favorable influence is exerted on the disease. The remissions are sometimes so imperfect that even at this time there is a tendency to stupor, and in one case that I treated in 1842 the patient lay eight days without speaking. When called to him he presented all the symptoms of apoplexy, and nothing revealed the true nature of the case but a disposition to yawn and stretch every morning, continuing from 7 A.M. to 10 A.M., and a slight abatement in the force, and a diminution of a few beats in the frequency of the pulse, with a temporary disappearance of the stertor. During the remissions, while yawning and stretching, his appearance was exactly that of a person just awaking from a sound and refreshing sleep, and the bystanders, even those who had seen him several times, could scarcely divest themselves of the impression that this was the case, and were in momentary expectation of seeing him open his eyes and address them. The case terminated favorably, the patient waking up during the hour of remission on the 9th morning, and required but little treatment after."

In the neighborhood of this city cases of comatose remittents and intermittents are occasionally seen, the termination of which, now and then, is in sudden death. Patients have been known to suffer for several days with slight paroxysms of fever, hardly keeping their beds, and deeming medical aid almost unnecessary, and then, at the access of the chill, fall into an apoplectic state and sink in a few hours. Ordinary observers would find some difficulty in distinguishing such cases from apoplexy; but the state of the skin and pulse reveals their true character to the medical man.

This paper is creditable to the industry, zeal and talents of the author, and will advance his reputation as a physician and a writer.

